# Long-term effectiveness of benralizumab in severe eosinophilic asthma patients treated for 96-weeks: data from the ANANKE study

**DOI:** 10.1186/s12931-023-02439-w

**Published:** 2023-05-20

**Authors:** Alessandra Vultaggio, Maria Aliani, Elena Altieri, Pietro Bracciale, Luisa Brussino, Maria Filomena Caiaffa, Paolo Cameli, Giorgio Walter Canonica, Cristiano Caruso, Stefano Centanni, Maria D’Amato, Fausto De Michele, Stefano Del Giacco, Fabiano Di Marco, Francesco Menzella, Girolamo Pelaia, Paola Rogliani, Micaela Romagnoli, Pietro Schino, Gianenrico Senna, Marco Benci, Silvia Boarino, Jan Walter Schroeder

**Affiliations:** 1 Dipartimento di Medicina Sperimentale e Clinica, Università degli Studi di Firenze, Florence, Italy; 2 UO Pneumologia e Pneumologia Riabilitativa, ICS Maugeri, IRCCS Bari, Bari, Italy; 3 Reparto di Pneumologia, P.O. Garbagnate, Milanese, Italy; 4 Reparto di Pneumologia, Ospedale Ostuni, Ostuni, BR Italy; 5 Dipartimento di Scienze Mediche, SSDDU Allergologia e Immunologia Clinica, Università degli Studi di Torino, AO Ordine Mauriziano Umberto I, Torino, Italy; 6 Cattedra e Scuola di Allergologia e Immunologia Clinica, Dipartimento di Scienze Mediche, Università di Foggia, Foggia, Italy; 7 Respiratory Diseases and Lung Transplantation, Department of Medical and Surgical Sciences and Neurosciences, Siena University Hospital, Siena, Italy; 8 Department of Biomedical Sciences, Humanitas University, Pieve Emanuele, MI Italy; 9 Personalized Medicine Center: Asthma and Allergology, Humanitas Research Hospital, Rozzano, MI Italy; 10 Dipartimento di Scienze Mediche e Chirurgiche, Fondazione Policlinico A. Gemelli, IRCCS, Università Cattolica del Sacro Cuore, Rome, Italy; 11 Respiratory Unit, ASST Santi Paolo e Carlo, Department of Health Sciences, Università degli Studi di Milano, Milan, Italy; 12 UOSD Malattie Respiratorie “Federico II”, Ospedale Monaldi, AO Dei Colli, Naples, Italy; 13 UOC Pneumologia e Fisiopatologia Respiratoria, AORN A. Cardarelli, Naples, Italy; 14 Department of Medical Sciences and Public Health, University of Cagliari, Cagliari, Italy; 15 Department of Health Sciences, Università degli Studi di Milano, Pneumologia, ASST Papa Giovanni XXIII, Bergamo, Italy; 16 UOC Pneumologia, Ospedale “S. Valentino”, AULSS 2 Marca Trevigiana, Montebelluna, TV Italy; 17 Dipartimento di Scienze della Salute, Università Magna Graecia, Catanzaro, Italy; 18 Division of Respiratory Medicine, University Hospital “Tor Vergata”, Rome, Italy; 19 Unit of Respiratory Medicine, Department of Experimental Medicine, University of Rome “Tor Vergata”, Rome, Italy; 20 UOC Pneumologia, AULSS 2 Marca Trevigiana, Treviso, Italy; 21 Fisiopatologia Respiratoria, Ospedale Generale Regionale, Ente Ecclesiastico “F. Miulli”, Acquaviva delle Fonti, BA Italy; 22 Department of Medicine, University of Verona, Verona, Italy; 23 Allergy Unit and Asthma Center, Verona University Hospital, Verona, Italy; 24 Medical Affairs R&I, AstraZeneca, Milan, Italy; 25 Medical Evidence R&I, AstraZeneca, Milan, Italy; 26 Allergy and Clinical Immunology, ASST Grande Ospedale Metropolitano Niguarda, Milan, Italy

**Keywords:** Benralizumab, Asthma, Eosinophils, Exacerbations, Long-term

## Abstract

**Background:**

The efficacy of benralizumab has been broadly demonstrated in severe eosinophilic asthma (SEA), but only few real-life studies evaluated its long-term effects. Here we present novel data from the ANANKE study in which a large cohort of SEA patients was treated for up to 96 weeks.

**Methods:**

ANANKE (NCT04272463) is an observational retrospective Italian study investigating the key characteristics of SEA patients (collected during the 12 months prior to benralizumab initiation) and the clinical outcomes during benralizumab treatment (annual exacerbation rate [AER], lung function, asthma control, OCS use, healthcare resource utilization). A post hoc analysis was also conducted in groups of patients based on history of previous biologic therapy (bio-experienced versus naïve patients). Analyses were descriptive only.

**Results:**

Before benralizumab initiation, evaluable SEA patients (N = 162, 61.1% females, mean age 56.0 ± 12.7) showed a median blood eosinophil count (BEC) of 600 cells/mm^3^ (IQR: 430–890). Patients experienced frequent exacerbations (annualized exacerbation rate [AER]: 4.10, severe AER: 0.98), with impaired lung function and poor asthma control (median ACT score: 14) despite 25.3% reported oral corticosteroid (OCS) use. Nasal polyposis was present in 53.1% patients; 47.5% patients were atopic. After 96 weeks since the start of benralizumab, nearly 90% patients were still on treatment; benralizumab dramatically decreased exacerbations (AER: − 94.9%; severe AER: − 96.9%), improved respiratory parameters (median increase in pre-bronchodilator forced expiratory volume [pre-BD FEV1]: + 400 mL) and asthma control (median ACT score: 23) while eliminating OCS in 60% patients. Importantly, benralizumab effects were either maintained or progressively improved over time, accompanied by a nearly complete depletion of BEC. Benralizumab reduced AER both in naïve (any AER: − 95.9%; severe AER: − 97.5%) and bio-experienced patients (any AER: − 92.4%; severe AER: − 94.0%).

**Conclusions:**

Profound and sustained improvements in all asthma outcomes were observed with benralizumab. The correct identification of patients’ eosinophilic-driven asthma phenotype was essential to ensure the achievement of such remarkable results.

*Trial registration*: ClinicalTrials.gov Identifier: NCT04272463.

**Supplementary Information:**

The online version contains supplementary material available at 10.1186/s12931-023-02439-w.

## Background

Severe eosinophilic asthma (SEA) patients are characterized by uncontrolled asthma, severe exacerbations and poor lung function that progressively declines over time [1–3]. The pathophysiology of SEA is distinctly driven by eosinophilic inflammation, reflected by a general but still not well-defined increase in blood and sputum eosinophil counts; in addition, SEA patients can present atopy and show an increment in other type 2 (T2) inflammatory biomarkers [4]. Oral corticosteroids (OCS) are often used to mitigate SEA; although they have been proved highly effective, the risk of developing OCS-associated adverse events and systemic chronic condition is nowadays well-accepted [5–10].

Benralizumab is an anti-interleukin 5 receptor (IL5R) monoclonal antibody (mAb) that holds an extra pro-apoptotic function gained through a specific mechanism of Ab-dependent cell-mediated cytotoxicity (ADCC) [11, 12]; as a result, benralizumab almost completely depletes the pool of IL5R high-expressing cells, namely eosinophils and basophils [13, 14]. Because of its potent anti-eosinophilic action, benralizumab is approved for the treatment of uncontrolled SEA, showing a broad efficacy in decreasing exacerbations and improving respiratory function while minimizing OCS use and asthma control, as demonstrated across a number of randomized controlled trials and real-life studies [15–20].

ANANKE (NCT04272463) [21] is an observational retrospective Italian study conducted on a large SEA population, aiming to examine patients’ clinical characteristics before starting benralizumab treatment, and benralizumab effectiveness. The main features of the ANANKE population previously described by Menzella and colleagues were consistent with the eosinophilic phenotype (median blood eosinophil count [BEC] > 550 cells/mm^3^, over 50% patients with current or past nasal polyposis, frequent exacerbations and compromised respiratory function with over 25% OCS users) [22]. Benralizumab treatment was shown to induce over 90% reduction in annualized exacerbation rate (AER) (both severe and any kind of exacerbations), with a concomitant reduction of OCS dosage of 56%, observed over a median period of 9.8 months. Patients also experienced an overall improvement in lung function and asthma control, resulting in a decreased utilization of healthcare resources [22]. Additional post hoc analyses were carried out and revealed that benralizumab maintained its efficacy independently of presence of atopy, body mass index (BMI) [22], presence of nasal polyps [23], use of previous biologics [24] and BEC [25].

Here we present novel data from the ANANKE study related to an extended treatment period (up to 96 weeks). To our knowledge, few real-life studies have been conducted on a large SEA population treated with benralizumab for longer than a year. Three long-term studies (mean of 19.7 months [18], 2 years [26] and up to 4 years [19]) examined a smaller number of patients and revealed that benralizumab effectiveness was maintained over time [18, 19, 26]. Thus, these data will add valuable information about benralizumab durability of action and further corroborate the eosinophilic-driving pathophysiological mechanism underlying SA in the ANANKE population.

## Material and methods

### Study design

ANANKE is an Italian multi-center, observational, retrospective study (ClinicalTrials.gov Identifier: NCT04272463) [21] in which SEA patients treated with benralizumab as per clinical practice or within the Italian Sampling Program were enrolled. As described in Menzella *et al.* [22], the “index date” represented the start date of benralizumab treatment and the enrolment visit occurred at least 3 months after the index date. Compared with the original study design, the observation period after the index date was extended from a period of at least 3 months to a period up to 96 weeks, as authorized by additional and amended study informed consent and privacy forms, which were signed by each patient. As a result, data were collected over a period of at least 15 months (i.e., during 12 months prior to index date and during a period ranging from 3 months to 96 weeks after the index date). During the observation period, data were collected at 4, 16, 24, 48 and 96 weeks after the index date, if available from patients’ hospital medical charts (Additional file 6: Fig. S1). ANANKE was performed in accordance with the principles of the Declaration of Helsinki and laws and guidelines regulating the Italian medical practice. Ethical approval was provided by the ethics committees/institutional review boards at each participating site.

### Study population

Inclusion and exclusion criteria have been extensively described elsewhere [22]. Briefly, the ANANKE study included adult patients (≥ 18 years old), diagnosed with SEA and requiring constant treatment with high doses of inhaled corticosteroids (ICS) and a long-acting b2-agonist (LABA) ± additional asthma controllers, who started treatment with benralizumab at least 3 months before enrolment (either within the sampling program or as per routine practice), had at least one benralizumab injection and had hospital medical charts available from 12 months prior to the index date. Patients were excluded from the ANANKE study if, after the enrollment visit, they participated in studies dictating a specific patient’s management strategy that differed from the site’s normal clinical practice.

Patients were considered eligible if they satisfied all inclusion, exclusion, and amendment criteria.

Evaluable patients included all eligible patients with available BEC data at index date.

### Outcomes and variables

Data were collected from hospital medical charts according to clinical practice and recorded in the electronic case report form (eCRF).

The primary endpoint aimed to outline patients’ characteristics at index date (i.e., the start of benralizumab treatment), which were collected during the 12 months prior to benralizumab introduction. Those included: patients’ demographics (age, sex), data obtained from physical examination (BMI), lifestyle habits (smoking status), aspects related to the onset of asthma (age at asthma diagnosis, asthma and SEA duration), laboratory assessments (BEC and total serum immunoglobulin E [IgE]), presence of atopy (defined as a positive skin prick test for a perennial and/or seasonal allergen), comorbidities (asthma-related and OCS-related conditions, any other relevant condition as per investigator’s opinion) lung function parameters, asthma control (assessed via the Asthma Control Test [ACT]), current and previous asthma medications (maintenance treatments, OCS use and dosage at the index date and any biologics used during the 12 months before the index date), annualized exacerbation rate (AER) for any exacerbation and severe exacerbation. Any exacerbation was defined as a physician-diagnosed clinically relevant asthma exacerbation, while severe exacerbation was considered as an exacerbation that worsened the asthma condition leading to one of the following: (a) use of systemic corticosteroids for 3 days or more or a temporary increase in background dosage of OCS; (b) an emergency department or urgent care visit (< 24 h) that required systemic corticosteroids; or c) an inpatient admission to hospital (≥ 24 h).

Secondary endpoints aimed to describe clinical outcomes during benralizumab treatment between the index date and 96 weeks, including data at 4, 16, 24, 48 and 96 weeks, if available from patients’ hospital medical charts. In particular, the following parameters were evaluated:

1. Change in BEC as a consequence of benralizumab mechanism of action (MoA);
2. Change in AER (any and severe exacerbations) and proportion of patients free from any and severe exacerbations;
3. Change in ACT score, proportion of patients with ACT ≥ 20 and proportion of patients achieving minimal clinical important difference (MCID, defined as a change accounting for ≥ 3 points with respect to index date);
4. Change in lung function (pre- and post- bronchodilator [BD] forced expiratory volume in the first second [FEV_1_], forced vital capacity [FVC]);
5. Change in OCS use and daily dosage (expressed as prednisone-equivalent mg), proportion of patients eliminating OCS usage.

Other outcomes that were assessed included: healthcare resources utilization, adherence, persistence and discontinuation to benralizumab treatment. Adherence was measured as the ratio between the number of actual injections and the number of expected injections at 96 weeks (in percentage); persistence was evaluated using the Kaplan–Meier survival analysis. A *post hoc* analysis was conducted to describe differences in demographic and clinical characteristics between patients who were naïve to biologic treatments compared to patients who switched from other biologics (omalizumab and mepolizumab) because of suboptimal response (biologic-experienced patients). Such differences were described both at index date and during benralizumab treatment (up to 96 weeks). Clinical outcomes measured during benralizumab treatment included AER (any and severe) and asthma control, assessed using the ACT questionnaire.

### Statistical analysis

Statistical analyses were extensively described in Menzella et al. [22]. Briefly, no formal hypothesis was prespecified and analyses were descriptive only. Data are expressed as either mean, standard deviation (SD), median, interquartile range (IQR) or absolute numbers and relative frequencies as appropriate. Median and IQR were used instead of mean (SD) in case of a highly variable distribution of data.

Demographic and clinical characteristics at the index date are presented for all evaluable patients (N = 162); secondary and exploratory endpoints were assessed in evaluable patients for secondary analyses at 48 (N = 145) and 96 weeks (N = 113); data related to adherence, persistence, and discontinuation to benralizumab treatment were evaluated in eligible patients with consistent data at 96 weeks (N = 115).

Analyses were carried out using SAS software v9.4 (SAS Institute, Cary, NC, USA).

## Results

### Patients disposition

As previously detailed [22], 218 SEA patients were enrolled between December 2019 and July 2020 from 21 centers in Italy to take part in the ANANKE study. Of those, 167 patients (76.6%) were considered eligible for analysis as per inclusion/exclusion criteria. Of the 51 non-eligible patients, 2 patients did not start benralizumab treatment at least three months before enrollment, 46 patients failed to fill and/or sign the informed consent and privacy (amendment) forms; 3 patients had no ongoing treatment with ICS and LABA at the index date. Eligible patients with consistent data at 48 and 96 weeks were 150 and 115, respectively. A total of 5 patients were considered non-evaluable because of missing BEC values at the index date. Therefore, evaluable SEA patients were 162 and patients considered evaluable for secondary analyses at 48 and 96 weeks were 145 and 113, respectively (Additional file 1: Table S1). Of note, not all patients were evaluable for all parameters at both 48 and 96 weeks.

### Characteristics are consistent with the severe eosinophilic asthma phenotype.

Demographic, clinical and laboratory characteristics of evaluable patient at 96 weeks are shown in Table 1. Additional information related to maintenance asthma therapies, positivity to perennial allergens, comorbidities and OCS-related conditions are detailed in Additional file 2: Table S2. Data were collected before the start of benralizumab therapy (at the index date or during the 12 months prior to the index date). Briefly, patients (mean age: 56.0 ± 12.7 years) were predominantly females (N = 99; 61.1%), and non-smokers (N = 106; 65.4%). Additionally, approximately 40% patients were overweight (N = 62; 38.3%). Median asthma duration was 13.5 years (IQR 8.1–25.4) and median time since SEA diagnosis was 1.8 years (IQR 1.0–4.3). A total of 77 patients (47.5%) were positive for perennial and/or seasonal allergen; among asthma-related comorbidities, nasal polyposis (current or past) was the most frequent (N = 86; 53.1%). Median BEC was 600 cells/mm^3^ (IQR 430–890) and total serum IgE were 215 IU/ml (IQR 83–520). At index date, 41 patients (25.3%) used OCS chronically, with a median daily dose (prednisone-equivalent) of 10 mg (IQR 5–25, data from N = 39). During the 12 months prior to benralizumab initiation, other biologic therapies (omalizumab and mepolizumab) were administered to 38 patients (23.5%).

**Table 1:** Socio-demographic, clinical and laboratory characteristics of patient population collected before initiating benralizumab treatment

Characteristics at index date	Evaluable population (N = 162)
Age (years)	56.0 ± 12.7
Females	99 (61.1)
BMI (kg/m^2^)	
Underweight/normal weight Overweight Obese Unknown	58 (35.8) 62 (38.3) 26 (16.0) 16 (9.9)
Smoking status	
Current smokers Past smokers	5 (3.1) 44 (27.2)
Asthma duration (years) (N = 161)	13.5 (8.1–25.4)
SEA duration (years) (N = 158)	1.8 (1.0–4.3)
Patients positive to ≥ 1 (perennial and/or seasonal) allergen	77 (47.5)
BEC (cells /mm^3^) Total serum IgE (IU/mL)	600 (430–890) 215 (83–520)
Comorbidities	141 (87.0)
≥ 1 current asthma-related condition Nasal polyposis, current or past Chronic rhinosinusitis without nasal polyposis ≥ 1 current OCS-related condition (osteoporosis, type 2 diabetes etc.) ≥ 1 other ongoing comorbidities	91 (56.2) 86 (53.1) 43 (26.5) 64 (39.5) 38 (23.5)
OCS for asthma treatment OCS daily (prednisone equivalent) dose (mg) (N = 39)	41 (25.3) 10 (5–25)
Exacerbations during the 12 months prior to index date (N = 154)	
Patients with ≥ 1 exacerbation of any severity Patients with ≥ 1 severe exacerbation	144 (93.5) 57 (37.0)
AER, any	4.10
AER, severe	0.98
Lung function	
Pre-BD FEV1 (L) (N = 121) Post-BD FEV1 (L) (N = 80) Pre-BD FEV1 predicted (%) (N = 125) Post-BD FEV1 predicted (%) (N = 79) Pre-BD FVC (N = 116) Post-BD FVC (N = 75) FeNO (ppb) (N = 52)	1.9 (1.4–2.5) 2.0 (1.4–2.8) 71.0 (54.0–84.0) 73.0 (59.0–93.0) 2.8 (2.3–3.5) 3.0 (2.3–3.9) 42.0 (23.0–66.0)
ACT score (N = 120)	14 (12–17.5)
Patients previously treated with biologics	38 (23.5)
Healthcare resource utilization for asthma per patient (N = 150)	
Primary care physician/GP office visits Specialist visits ED admissions Hospitalizations	1.1 ± 1.8 2.4 ± 2.5 0.1 ± 0.4 0.2 ± 0.5

Data were collected at the index date or during the 12 months prior to the index date and are expressed as N (%), mean ± SD, or median (IQR). Unless otherwise stated, the evaluable population included 162 patients

Despite the use of asthma medications, patients’ asthma was not controlled. As a matter of fact, during the year before benralizumab initiation, 144 out of 154 patients (93.5%) experienced at least one asthma exacerbation of any severity, while 57 patients (37%) experienced at least one severe exacerbation. Any and severe AER were respectively 4.10 and 0.98. Patients were also characterized by suboptimal lung function (median pre-BD FEV_1_ predicted: 71%, IQR 54–84) and low ACT score (median ACT score: 14; IQR 12–17.5). Because of uncontrolled asthma, patients most frequently attended specialist visits (mean visits per year: 2.4 ± 2.5); occasionally, patients were either hospitalized (mean hospitalizations per year: 0.2 ± 0.5) or admitted to ED (mean ED admissions per year: 0.1 ± 0.4).

Overall, the characteristics described here largely overlap the features already reported by Menzella et al. [22] and confirm the severe eosinophilic nature of asthma in the ANANKE population.

### Exposure, persistence, adherence and discontinuation to benralizumab treatment

The median exposure to benralizumab treatment was 98.4 weeks (IQR 94.7–104). At 96 weeks, 103 out of 115 eligible patients with consistent data (89.6%) were still on treatment with benralizumab; patients receiving more than 90% of expected injections were 96 (83.5%) with an average number of 13.0 ± 1.6 injections per patient. On average, the level of adherence to benralizumab treatment was 95.1% (± 10.4). Benralizumab was permanently discontinued in 12 patients (10.4%); Additional file 7: Fig. S2 represents the time from index date to treatment discontinuation (weeks). The reasons for benralizumab interruption were lack of clinical efficacy (N = 8, 66.7%) and patient decision (N = 2, 16.7%); two patients discontinued for unknown reasons (16.7%). Of these, 7 patients received alternative biologic treatments (omalizumab or dupilumab).

### Benralizumab maintained long-term depletion of blood eosinophils and minimized exacerbations

**Fig. 1:**
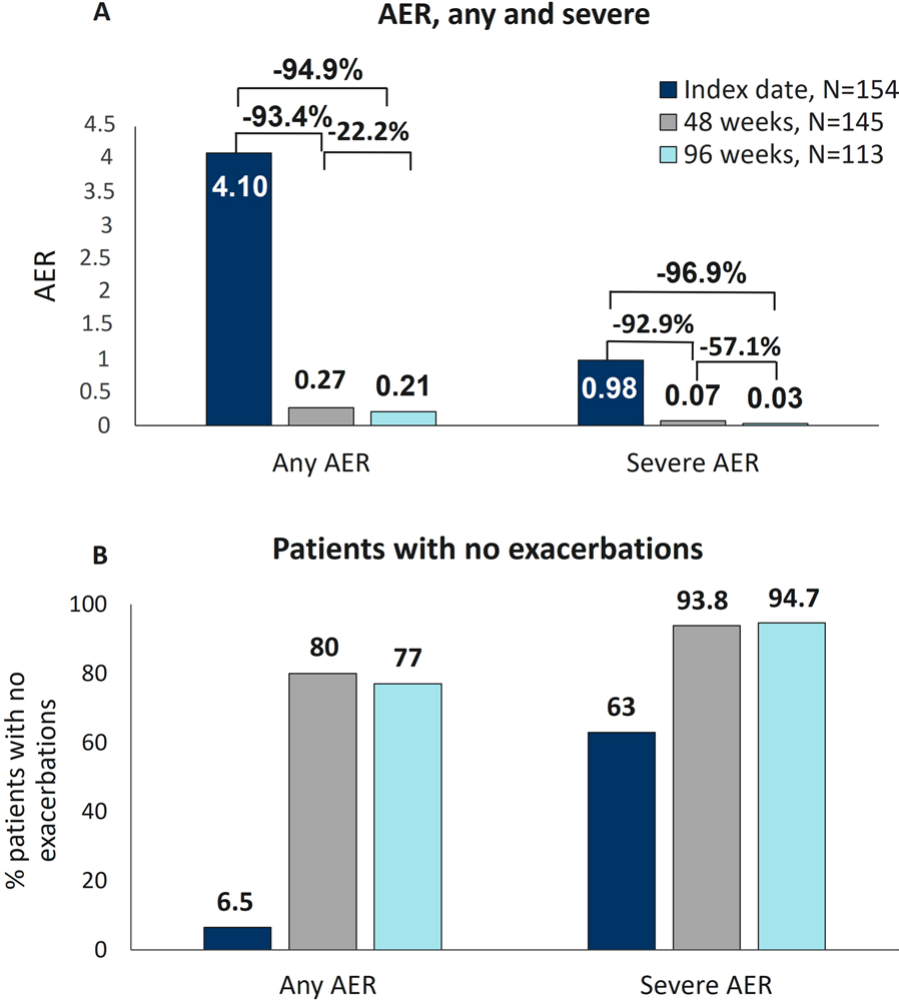
Sustained reduction in exacerbations during benralizumab treatment. Any and severe AER (**A**) and the percentage of patients without any exacerbations (**B**) are shown at index date and after 48 and 96 weeks of treatment with benralizumab

Consistently with its mechanism of action, benralizumab induced a nearly complete depletion of BEC that was already observed at 16 weeks as previously reported [22] and was sustained throughout the 96-week treatment period (median BEC: 0.0 cells/mm^3^, IQR 0.0–0.0) (Additional file 8: Fig. S3). During this time frame, benralizumab dramatically decreased the frequency of exacerbations; compared with the index date, any AER was reduced by 93.4% (4.10 vs 0.27) at 48 weeks and 94.9% (4.10 vs 0.21) at 96 weeks, with a reduction of 22.2% occurring in the period between 48 and 96 weeks (0.27 vs 0.21, respectively). Likewise, severe AER decreased by 92.9% (0.98 vs 0.07) at 48 weeks with a further reduction of 96.9% (0.98 vs 0.03) observed at 96 weeks from the index date, with a 57.1% reduction observed between 48 and 96 weeks; 0.07 vs 0.03, respectively) (Fig. 1A). As expected, an elevated proportion of patients reported zero exacerbations; at 96 weeks, 87 out of 113 patients (77%) did not experience any kind of exacerbations and 107 out of 113 (94.7%) were free from severe exacerbations (Fig. 1B). Healthcare resource utilization (specifically related to asthma) was quantified throughout the ANANKE study; in line with previous results [22], an average of 0 ER admissions and 0 hospitalizations were recorded per patient at 96 weeks (0.0 ± 0.2 and 0.0 ± 0.1, respectively). The number of primary care physician/GP visits and specialistic visits also dropped from index date to 96 weeks (1.1 ± 1.8 vs 0.1 ± 0.6 and 2.4 ± 2.9 vs 1.0 ± 2.2, respectively) (Additional file 3: Table S3).

### Benralizumab preserved lung function with long-term improvements of pre- and post-BD FEV_1_ and FVC

**Fig. 2:**
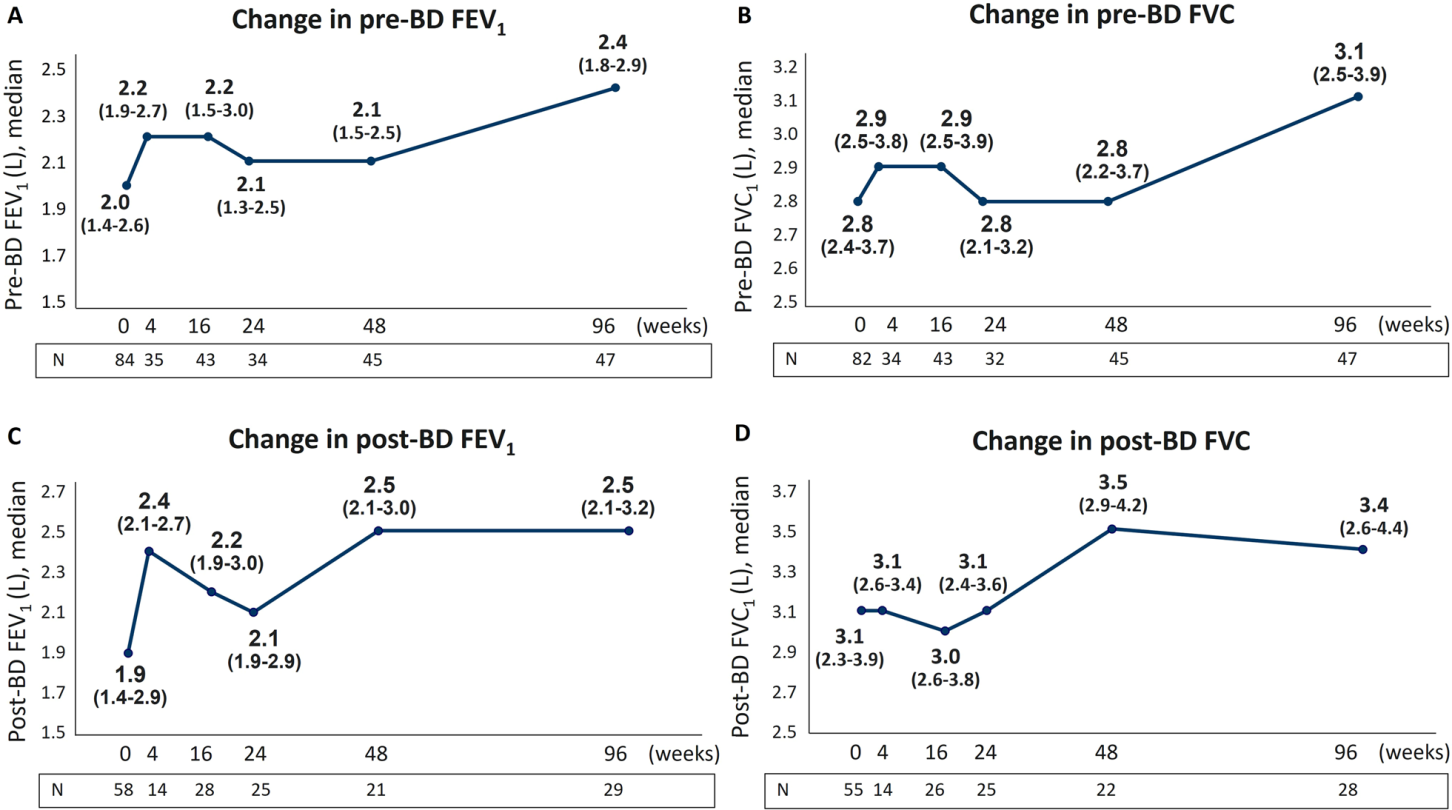
Long-term effect of benralizumab on lung function. FEV_1_ (**A**, **C**) and FVC (**B**, **D**) values pre- and post-BD are shown at various time points (index date and 4, 16, 24, 48, 96 weeks of treatment with benralizumab)

As shown in Fig. 2, pre-BD FEV_1_ values were overall increased from index date, with an effect already noticeable at 4 weeks and a sharp increase becoming evident at 96 weeks (from 2.0 L to 2.4 L, + 400 mL) accompanied by a similar increase in pre-BD FVC (from 2.8 L to 3.1 L, + 300 mL) (Fig. 2A, B). Post-BD FEV_1_ and FVC values showed a major increase at week 48 (post-BD FEV_1_: from 1.9 L to 2.5 L, + 600 mL; post-BD FVC: from 3.1 L to 3.5 L, + 400 mL) which was maintained until 96 weeks (Fig. 2C, D).

### Benralizumab induced a continuous, long-term improvement in asthma control

**Fig. 3:**
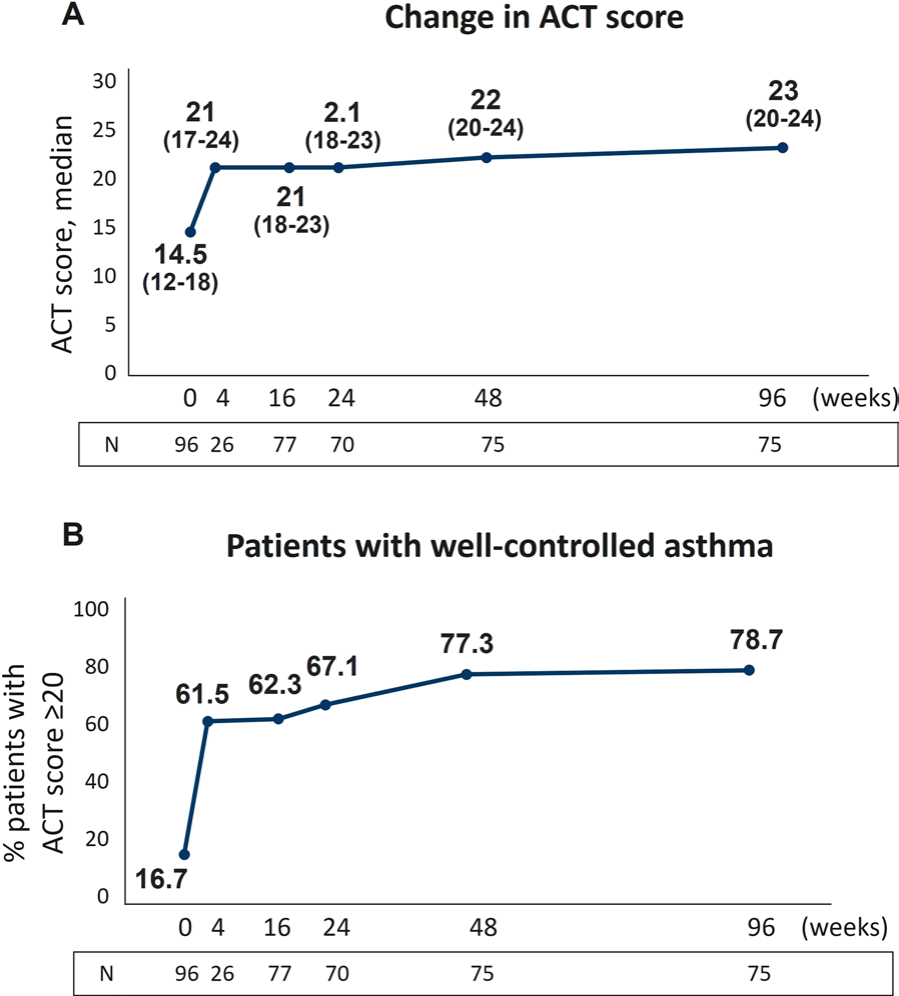
Continuous improvement in asthma control during benralizumab treatment. **A** Change in ACT score is expressed as median (IQR). **B** Proportion of patients achieving well-controlled asthma (ACT score ≥ 20). Data are reported at index date and after 4, 16, 24, 48 and 96 weeks of treatment with benralizumab

In agreement with the beneficial effects observed on exacerbation rate and lung function parameters, benralizumab considerably improved asthma control as assessed by ACT questionnaire. Figure 3A shows that the median ACT score progressively increased from index date, reaching the highest value at 96 weeks with a median increase of 8.5 points (from 14.5 to 23). The proportion of patients achieving a well-controlled asthma (ACT score ≥ 20) also increased from 16.7% at index date with a peak of 78.7% patients at 96 weeks (Fig. 3B). Importantly, these changes were already evident at 4 weeks, when the median ACT score reached a value of 21 and 61.5% patients already reported complete asthma control. MCID in ACT score was achieved by more than 62% patients at all time points considered (4,16, 24, 48, 96 weeks), with 73.1% patients obtaining MCID at 96 weeks (Additional file 4: Table S4).

### Long-term treatment with benralizumab promoted OCS elimination

**Fig. 4:**
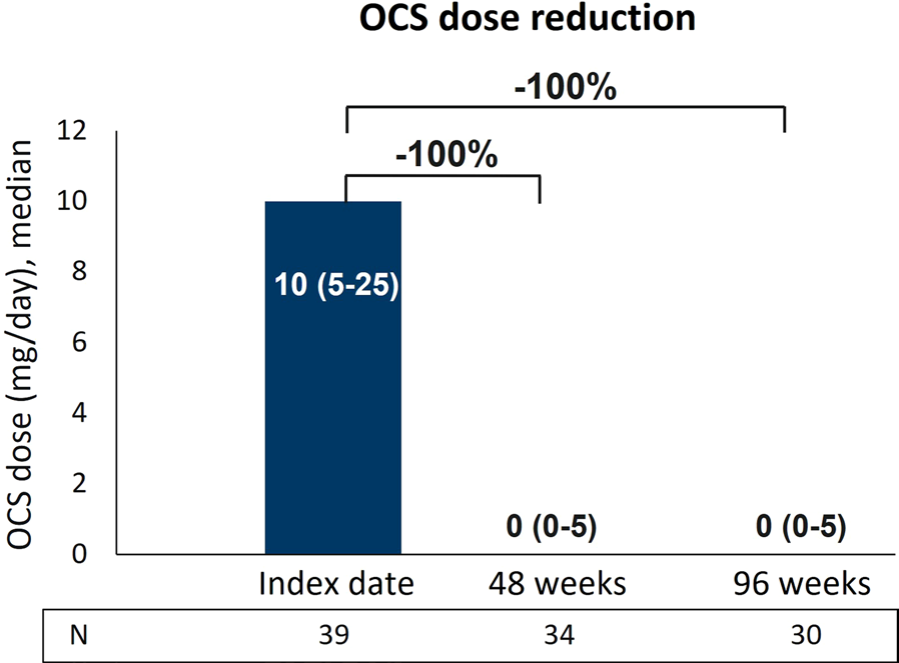
Sustained reduction in OCS usage during benralizumab treatment. Daily OCS dosage (mg of prednisone equivalent), expressed as median (IQR), is shown at index date and after 48 and 96 weeks of treatment with benralizumab

Benralizumab reduced OCS median daily dosage by 100% (0 mg, IQR 0–5) at both 48 and 96 weeks (Fig. 4). Overall, OCS dosage was decreased in 21 out of 34 patients (61.8%) and 20 out of 30 patients (66.7%) at 48 and 96 weeks respectively, with the majority of patients achieving a reduction equal or greater than 90% of the initial dose (Table 2). A total of 18 out of 34 patients (52.9%) completely dismissed OCS use by 48 weeks; importantly, OCS interruption was maintained at 96 weeks (18 out of 30 patients, 60%) (Fig. 4; Table 2). These data further improve the results described in Menzella et al., where the median OCS daily dosage was decreased to 4.4 mg daily (prednisone equivalent), corresponding to 56% median reduction, and OCS were interrupted in 43.2% patients [22].

**Table 2:** OCS dose reduction from benralizumab treatment initiation to 96 weeks of treatment

Variable	Number (%) of patients with OCS reduction at 48 weeks (N = 34)	Number (%) of patients with OCS reduction at 96 weeks (N = 30)
OCS interruption	18 (52.9)	18 (60.0)
Any OCS reduction (including interruption)	21 (61.8)	20 (66.7)
≥ 90% OCS dose reduction	18 (52.9)	18 (60.0)
≥ 75% OCS dose reduction	18 (52.9)	18 (60.0)
≥ 25% OCS dose reduction	20 (58.8)	19 (63.3)
No OCS reduction	13 (38.2)	10 (33.3)

OCS dosage is expressed as daily mg (prednisone equivalent). Evaluable patients for secondary analyses at 48 and 96 weeks with OCS dose available were considered (N = 34 and N = 30, respectively). Data are expressed as N (%)

### Post hoc analysis: benralizumab efficacy is consistent between naïve and bio-experienced patients 

Among the 38 patients (23.5%) treated with biologics before switching to benralizumab, 21 patients (13%) were treated with omalizumab, 13 patients (8%) received mepolizumab and 4 patients (2.5%) switched from omalizumab to mepolizumab. The median duration of treatment with biologics prior to benralizumab initiation was 21.0 months (IQR 10.6–52.9); the median period during which patients interrupted biologics treatment before initiating benralizumab was 2.3 months (IQR 1.3–4.9) (data obtained from N = 29 patients). Demographic characteristics at index date were similar between naïve and bio-experienced patients, while clinical characteristics showed some differences between the two groups (Additional file 5: Table S5). In agreement with the post hoc data reported by Caruso et al. [24], naïve population had a shorter median duration of SEA (1.6 years naïve patients vs 3.5 years bio-experienced patients, IQR 1.0–3.0 vs 1.6–6.5, respectively), the bio-experienced group had a greater proportion of atopic patients (45.2% naïve patients vs 55.3% bio-experienced patients) and a higher frequency of OCS-related conditions (35.5% naïve patients vs 52.6% bio-experienced patients). Accordingly, OCS use appeared to be slightly higher in the bio-experienced group (24.2% naïve patients vs 28.9% bio-experienced patients) while BEC was slightly lower (median BEC: 605 cells/mm^3^ IQR 440–915 in naïve patients vs 550 cells/mm^3^ IQR 300–756 in bio-experienced patients). Importantly, while AER of any kind was comparable between the two groups (4.15 vs 3.95 in naïve and bio-experienced patients, respectively), severe AER was greater in bio-experienced patients (0.81 vs 1.51 in naïve and bio-experienced patients, respectively).

**Fig. 5:**
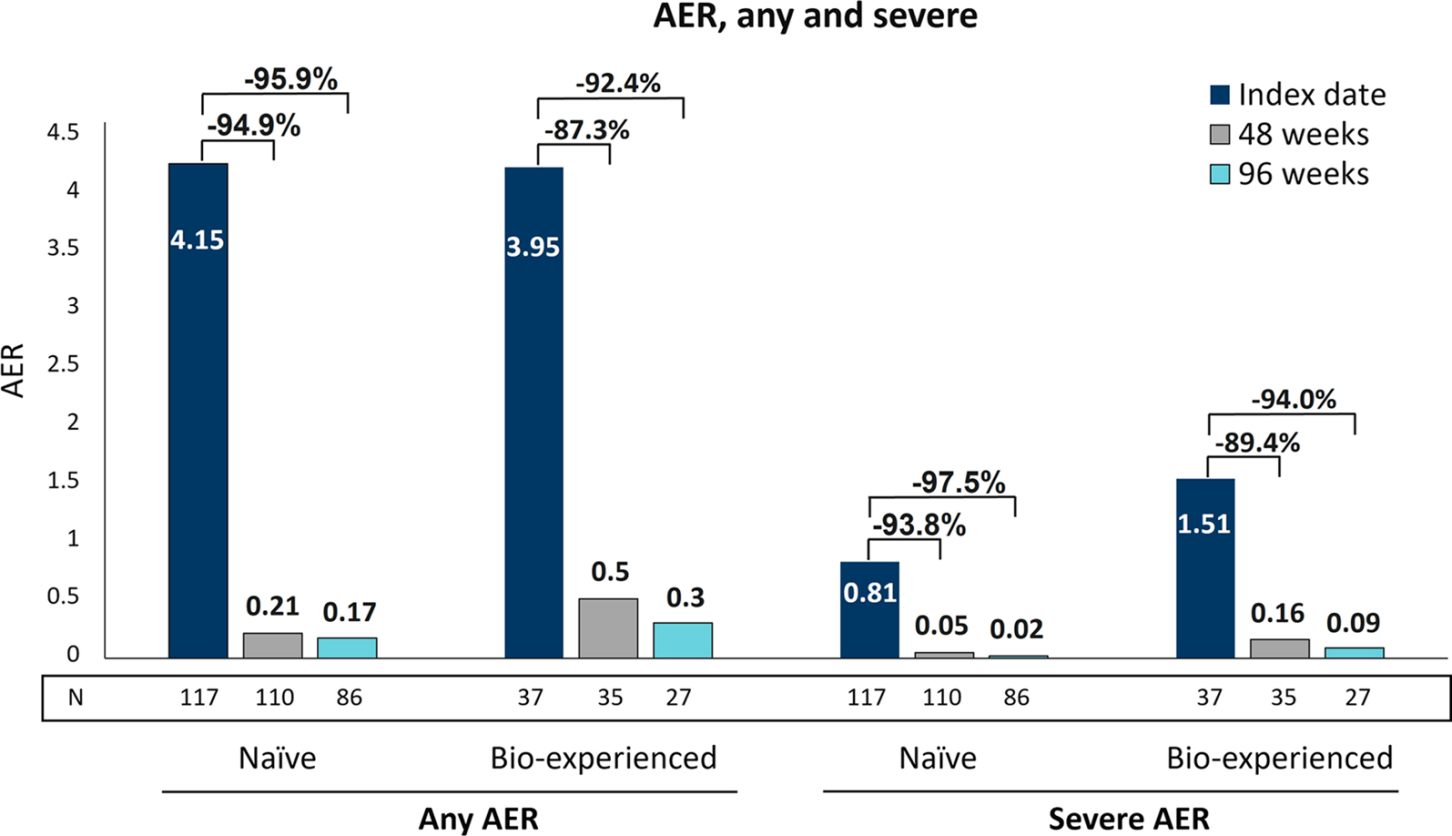
Benralizumab-induced AER reduction in naïve and bio-experienced patients. Any AER and severe AER are shown at index date and after 48 and 96 weeks after treatment with benralizumab

During the treatment period, benralizumab induced a pronounced effect in both naïve and bio-experienced populations. Any AER was almost completely eliminated in naïve patients, with reductions of 94.9% and 95.9% at 48 and 96 weeks, and an analogous decrease of 87.3% and 92.4% was noticeable in bio-experienced patients at 48 and 96 weeks, respectively. Severe AER was also declined in both naïve and bio-experienced patients with reductions of 97.5% (naïve) and 94.0% (bio-experienced) at 96 weeks (Fig. 5). Likewise, ACT score increased in both populations reaching median values of 23 (IQR 20–24) and 22 (IQR 19–24) at 96 weeks in naïve and bio-experienced patients, respectively (data not shown).

## Discussion

ANANKE (NCT04272463) [21] is one of the largest RWE studies conducted to examine the key features of SEA patients and the clinical outcomes achieved during benralizumab treatment. The data reported here complement previously published results from the ANANKE study, related to a shorter treatment period (median treatment duration: 9.8 months) [22].

As previously discussed [22], the clinical characteristics of the SEA population treated in the ANANKE study (diagnosis of asthma during adulthood, presence of circulating blood eosinophils, moderate airflow obstruction, more than half of the patients suffering from nasal polyposis and other asthma-related comorbidities, frequent exacerbations and a considerable proportion of OCS users) are consistent with the adult-onset, eosinophilic asthma phenotype. As already well-established [27, 28] benralizumab is highly effective in this subset of patients regardless of baseline BEC levels [25] and its effectiveness has been further corroborated in this study with novel long-term data. At 96 weeks, nearly 90% patients were still on treatment. This result is already indicative of the extensive beneficial effects induced by benralizumab, demonstrating that most patients had a positive experience and were willing to continue the treatment.

The frequency of asthma exacerbations represents the leading endpoint to evaluate how effective are therapies in alleviating the burden of severe asthma. The well-known efficacy of benralizumab in reducing exacerbations is strengthened in this study and its effectiveness is sustained over a prolonged period; at 96 weeks, AER of any severity and severe AER were decreased by 94.9% and 96.9%, respectively. Notably, 77% patients free from any exacerbations and 94.9% patients remained without severe exacerbations for the whole duration of the study. As previously reported by Menzella and colleagues, any and severe AER were already reduced by 93.3% and 94.5% after patients were treated for a shorter period (median treatment duration: 9.8 months) [22]. These results are also consistent with data obtained from the Phase III MELTEMI study, where exacerbations decreased progressively over the 5-year treatment period [15]; a similar drop of AER (from 4.1 to 0.33) has been found in the RWE study published by Sposato et al., in which 95 patients were treated for a mean period of 19.7 months [18]. In addition, a recent real-life study by Vitale and colleagues showed that benralizumab eliminated exacerbations in 85% patients (all OCS-dependent) after 2 years of treatment [26].

Exacerbation frequency is known to be associated with greater BEC [29]; in light of benralizumab exclusive MoA and the results presented here and elsewhere [15–19], the strong effect of benralizumab in reducing exacerbations could be considered a hint to unlock clinical remission in SEA.

Severe asthma patients typically experience a progressive deterioration in lung function over time [30]. Even if spirometric data were limited and not all patients were evaluated at all time points, our results suggest that overall, benralizumab treatment enhances FEV_1_ from the very first administration (4 weeks) and preserves lung function over time as demonstrated by spirometry parameters collected at various time points. While post-BD levels of FEV_1_ and FVC were increased at 48 weeks and remained stable up until 96 weeks, a sharp improvement in pre-BD FEV_1_ (change in median volume: + 400 mL) was evident only after 96 weeks of treatment.

Albeit additional measurements at later time points would be needed to confirm this result, we speculated this effect to be a direct consequence of benralizumab-induced sustained depletion of eosinophils within the lung tissue. As a matter of fact, our data showed a nearly complete depletion of BEC maintained over time, yet it is known that benralizumab has a similar effect in depleting eosinophils in sputum [31]. Moreover, exacerbation frequency is known to be associated with a more pronounced decline in lung function [32, 33]; consistently, we found exacerbations to be almost completely eradicated with a parallel amelioration in pre-BD parameters. Of note, FEV_1_ increased more than 300 mL in the 1-year long phase III SIROCCO and CALIMA studies and such level was maintained, but did not further improve, during the 1-year extension study BORA [34–36]. In general, the majority of RWE studies highlighted the rapid action of benralizumab in improving lung function [37, 38], with only few studies that investigated benralizumab long-term effects on multiple respiratory parameters. One of these studies reported a progressive improvement in FEV_1_, FVC and FEV_1_/FVC levels after 26 and 52 weeks of treatment in a small cohort of 18 SEA patients, with FEV_1_ increasing more than 1000 mL in patients with nasal polyposis and BEC > 500 cells/mm^3^ [39]. Three other studies assessed FEV_1_ over a period greater than 1 year [18, 19, 26]. The data published by Sposato and colleagues, showing an improvement of + 300 mL in FEV_1_ [18] are comparable with our results, yet the measurements were taken only at baseline and at the end of the study, hence it is difficult to determine if the improvement was continuous or limited to the first period of treatment. The multiple respiratory assessments performed at various time points in the study by Vitale et al. demonstrated a significant and stable increase in all respiratory parameters, with the greatest improvement detected within the first 6 months of treatment with benralizumab [26].

Asthma control was also enhanced, as assessed by median ACT score and proportion of patients achieving total control of asthma and MCID; notably, all these variables sharply increased at 4 weeks and continued to rise. ACT score has been extensively shown to be improved in the SEA population [18, 19, 26, 37, 38, 40], nevertheless the various time points assessed in our study reinforce both the rapidity and the durability of action of benralizumab in ameliorating patients’ asthma control.

To manage asthma-related symptoms and avoid exacerbations, SEA patients are often treated with OCS in a chronic manner. To date, a large body of literature supports a predominant role of benralizumab in sparing and even eliminating the use of OCS [16, 17, 20, 37, 38, 41, 42]. This effect is confirmed here, as benralizumab further decreased OCS dosage compared to the initial ANANKE analysis (in which OCS dosage decreased by a median of 56% with 43% patients who interrupted their use) and appears to be generally superior compared to the effect seen in other studies [16, 18, 19, 37, 41]. Here we describe a median OCS reduction of 100% achieved at 48 weeks and maintained at 96 weeks, accompanied by a parallel increase in the proportion of patients who discontinued OCS treatment. In this context, it is important to reiterate the impact of benralizumab on exacerbation rate, asthma control, and lung function in parallel with the concomitant elimination or reduction of OCS therapy.

SEA is associated with a substantial need of healthcare services and high healthcare-related costs; this is particularly true when patients experience frequent exacerbations [43]. Given the improvement in the asthma-related outcomes described above, it is not surprising to find that the average rate of emergency department admissions and hospitalization was eliminated after 96 weeks of benralizumab treatment.

Switching from one biologic therapy to another because of suboptimal asthma control is becoming more and more frequent thanks to the increasing number of mAbs currently approved for SA. The results from the post hoc analysis conducted here show a dramatic reduction in any and severe AER at 48 weeks, with a further decrease observed at 96 weeks, in both naïve and bio-experienced patients. Of note, the bio-experienced patients had a higher severe AER and a longer duration of severe asthma at index date, hence these data suggest that benralizumab is highly efficacious and ensures identical, maximal clinical outcomes even in patients in which previous biologic therapies failed to prevent exacerbations and improve asthma control.

Among the bio-experienced patients, the majority was previously treated with omalizumab (55.3%), with 34.2% patients receiving mepolizumab and a smaller proportion of patients switching from omalizumab to mepolizumab before being treated with benralizumab. Although it is difficult to predict which biologic treatment would be more efficacious in patients with multiple elevated T2 inflammatory biomarkers and there are no head-to-head studies directly comparing the various biologics, our data suggest that benralizumab ensures a considerable efficacy in patients with a true eosinophilic phenotype, where eosinophils play a major role in the progression and severity of the disease. In such patients, the choice between omalizumab and benralizumab would favor the latter, though the choice between mepolizumab and benralizumab would be more difficult, as both mAbs reduce eosinophils. Nevertheless, benralizumab is known to lower eosinophil count to a greater extent compared with mepolizumab both in blood and sputum, suggesting a superior effect in minimizing eosinophilic inflammation within the lung [44]. To date, a growing body of literature indicate that switching from mepolizumab to benralizumab further improves clinical outcomes in SEA patients [19, 45, 46], suggesting that a wider reduction of eosinophils may produce greater beneficial effects. Even though a direct comparison was not performed and baseline characteristics were different, the 1-year data obtained by Maglio et al. showed a greater percentage of benralizumab-treated patients that achieved clinical remission (defined as elimination of exacerbations and OCS, ACT ≥ 20 and FEV_1_ ≥ 80%) compared with patients treated with mepolizumab [47].

Because of the observational retrospective design of the ANANKE study, we could not formulate any formal statistical hypotheses thus the results are descriptive only. Other limitations are represented by the lack of a control group and the low number of patients that could be consistently evaluated at all time points. We also acknowledge that the prolonged period during which data were collected before benralizumab initiation (within 12 months prior to index date) may represent a significant bias for the homogeneity of baseline data. Nevertheless, we believe that the ANANKE data shown here will be highly informative for the scientific community and can positively impact daily clinical practice.

## Conclusions

In conclusion, the SEA population evaluated in the ANANKE study had a remarkable and durable response to benralizumab. The long-term improvements in all clinical outcomes, in combination with patients’ clinical characteristics at index date, underline a specific “eosinophilic-driven” asthma profile (as recently defined by Couillard et al.) [48] that features the ANANKE SEA population. Of note, the specific design of the ANANKE study, its retrospective nature (enrolment at least 3 months after benralizumab initiation) and the possibility to follow up patients until 96 weeks of treatment were pivotal to allow a thorough investigation of the patients’ key features as well as the achievement of such profound results. Once again, these data highlight that when benralizumab is administered to the right patient, with an authentic “eosinophilic-driven” asthma phenotype, it ensures a great, long-term efficacy thanks to the continuous and nearly complete depletion of eosinophils.

## Supplementary Information

**Additional file 1: Table S1**. Patients disposition. Data are expressed as N. Percentages computed over the total number of enrolled patients. Percentages computed out of the number of eligible patients.

**Additional file 2: Table S2**. Additional information related to maintenance asthma treatments, positivity to perennial allergens, comorbidities and OCS-related conditions collected before initiating benralizumab treatment. Data were collected at the index date or during the 12 months prior to the index date and are expressed as N, mean ± SD, or median. Unless otherwise stated, the evaluable population included 162 patients.

**Additional file 3: Table S3**. Asthma-related healthcare resource utilization during benralizumab treatment. Data are expressed as mean ± SD.

**Additional file 4: Table S4**. Patients achieving MCID in ACT total score during benralizumab treatment. Data are reported as number and percentage of patients achieving MCID.

**Additional file 5: Table S5**. Socio-demographic, clinical and laboratory characteristics, data on prior asthma medication, exacerbations, lung function, ACT score of naïve and bio-experienced patients, collected before initiating benralizumab treatment. Data were collected at the index date or during the 12 months prior to the index date and are expressed as N, mean ± SD, or median. Unless otherwise stated, data are related to N = 124 naïve patients and N = 38 bio-experienced patients. Data are expressed as N, mean ± SD, or median. Unless otherwise stated, the evaluable population included 124 naïve patients and N = 38 bio-experienced patients.

**Additional file 6: Figure S1.** ANANKE study design. The index date represents the initiation of benralizumab treatment; enrollment of patients started at least 3 months after the index date and lasted approximately 8 months. Socio-demographic and clinical characteristics were collected at the index date and during the 12 months prior to the index date. Patients were followed up for up to 96 weeks after the index date.

**Additional file 7: Figure S2.** Kaplan–Meier survival analysis curve showing persistence to benralizumab treatment. For this analysis, eligible patients with consistent data were considered. Time from index date to treatment discontinuationis the time between index date and the date of benralizumab permanent discontinuation. Patients who did not interrupt benralizumab during the observation period were censored at date of enrolment visit.

**Additional file 8: Figure S3.** BEC depletion during benralizumab treatment. Median BECwas evaluated at index date and at 16, 24, 48 and 96 weeks.

## Data Availability

The datasets used and/or analyzed during the current study are available from the corresponding author on reasonable request.

## Abbreviations

ACT: Asthma control test
ADCC: Afucosylation-dependent Ab-dependent cell-mediated cytotoxicity
AER: Annual exacerbation rate
BEC: Blood eosinophil count
BMI: Body mass index
COPD: Chronic obstructive pulmonary disease
eCRF: Electronic case report form
ED: Emergency department
FeNO: Fractional exhaled nitric oxide
GP: General practitioner
ICS: Inhaled corticosteroids
IgE: Immunoglobulin E
IL5R: IL5 receptor
IQR: Interquartile range
LABA: Long-acting β2-agonist
mAb: Monoclonal antibody
MCID: Minimal clinical important difference
OCS: Oral corticosteroids
pre/post BD-FEV_1_: pre/post bronchodilator-forced expiratory volume in 1 second
pre/post BD-FVC: pre/post bronchodilator-forced vital capacity
RWE: Real-world evidence
SD: Standard deviation
SEA: Severe eosinophilic asthma
