# Supplementary material for: Long-term effectiveness of benralizumab in severe eosinophilic asthma patients treated for 96-weeks: data from the ANANKE study
Source: Respir Res. 2023 May 20;24:135. doi: 10.1186/s12931-023-02439-w (PMC10200058; doi:10.1186/s12931-023-02439-w)
Supplement: Supplementary file 1 — Additional file 1: Table S1. Patients disposition. Data are expressed as N. Percentages computed over the total number of enrolled patients. Percentages computed out of the number of eligible patients. [file 12931_2023_2439_MOESM1_ESM.docx]

| **Patient disposition** | | **N (%)** |
| --- | --- | --- |
| **Enrolled** | | 218 (100.0) |
| **Eligible** (*) | | 167 (76.6) |
| Eligible with consistent data at 48 weeks (*) | | 150 (68.8) |
| Eligible with consistent data at 96 weeks (*) | | 115 (52.8) |
| **Evaluable** (**) | | 162 (97.0) |
| Evaluable for secondary analyses at 48 weeks (**) | | 145 (86.8) |
| Evaluable for secondary analyses at 96 weeks (**) | 113 (67.7) | |

**Supplementary table 1**
