# Supplementary material for: Long-term effectiveness of benralizumab in severe eosinophilic asthma patients treated for 96-weeks: data from the ANANKE study
Source: Respir Res. 2023 May 20;24:135. doi: 10.1186/s12931-023-02439-w (PMC10200058; doi:10.1186/s12931-023-02439-w)
Supplement: Supplementary file 2 — Additional file 2: Table S2. Additional information related to maintenance asthma treatments, positivity to perennial allergens, comorbidities and OCS-related conditions collected before initiating benralizumab treatment. Data were collected at the index date or during the 12 months prior to the index date and are expressed as N, mean ± SD, or median. Unless otherwise stated, the evaluable population included 162 patients. [file 12931_2023_2439_MOESM2_ESM.docx]

**Supplementary table 2**

| **Characteristics at index date** | **Evaluable population (N=162)**  **N (%)** |
| --- | --- |
| **Maintenance asthma treatment ongoing**  ICS/LABA  ICS + LABA  LAMA  Other (no OCS) | 160 (98.8)  2 (1.2)  83 (51.2)  76 (46.9) |
| **Patient positive for perennial allergens**  Dust mite (D. Pteronyssinus)  Cat hair  Dog hair  Aspergillus  Mould mix  Food mix  Other  **Other asthma-related comorbidities**  Gastroesophageal reflux (GERD)  Allergic rhinitis  Allergic conjunctivitis  Eosinophilic granulomatosis with polyangiitis (EGPA)  Chronic obstructive pulmonary disease (COPD)  Atopic dermatitis  Chronic idiopathic urticaria  Eosinophilic esophagitis | 63 (38.9)  45 (27.8)  15 (9.3)  13 (8.0)  12 (7.4)  5 (3.1)  3 (1.9)  5 (3.1)  39 (24.1)  38 (23.5)  26 (16.0)  9 (5.6)  3 (1.9)  1 (0.6)  1 (0.6)  1 (0.6) |
| **OCS-related conditions**  Hypertension  Osteoporosis  Cataract  Type 2 diabetes  Obstructive sleep apnoea  Cardiovascular disease  Anxiety/depression  Other | 38 (23.5)  17 (10.5)  10 (6.2)  9 (5.6)  8 (4.9)  7 (4.3)  7 (4.4)  18 (11.1) |
| **Other ongoing comorbidities** |  |
| Thyroid disorders | 7 (4.3) |
| Bronchiectasis | 6 (3.7) |
