# Supplementary material for: Long-term effectiveness of benralizumab in severe eosinophilic asthma patients treated for 96-weeks: data from the ANANKE study
Source: Respir Res. 2023 May 20;24:135. doi: 10.1186/s12931-023-02439-w (PMC10200058; doi:10.1186/s12931-023-02439-w)
Supplement: Supplementary file 3 — Additional file 3: Table S3. Asthma-related healthcare resource utilization during benralizumab treatment. Data are expressed as mean ± SD. [file 12931_2023_2439_MOESM3_ESM.docx]

**Supplementary table 3**

| **Healthcare service** | **Index date (N=150)** | **24 weeks (N=144)** | **48 weeks (N=139)** | **96 weeks (N=101)** |
| --- | --- | --- | --- | --- |
| Number of primary care physician/GP office visits per patient | 1.1 ± 1.8 | N/A | 0.0 ± 0.3 | 0.1 ± 0.6 |
| Number of specialist visits per patient | 2.4 ± 2.9 | N/A | 0.4 ± 1.4 | 1.0 ± 2.2 |
| Number of ER admissions per patient | 0.1 ± 0.4 | 0.0 ± 0.0 | 0.0 ± 0.3 | 0.0 ± 0.2 |
| Number of hospitalizations per patient | 0.2 ± 0.5 | 0.0 ± 0.0 | 0.0 ± 0.2 | 0.0 ± 0.1 |
