# Supplementary material for: Long-term effectiveness of benralizumab in severe eosinophilic asthma patients treated for 96-weeks: data from the ANANKE study
Source: Respir Res. 2023 May 20;24:135. doi: 10.1186/s12931-023-02439-w (PMC10200058; doi:10.1186/s12931-023-02439-w)
Supplement: Supplementary file 4 — Additional file 4: Table S4. Patients achieving MCID in ACT total score during benralizumab treatment. Data are reported as number and percentage of patients achieving MCID. [file 12931_2023_2439_MOESM4_ESM.docx]

**Supplementary table 4**

| **Period of treatment with benralizumab** | **Patient achieving MCID, N (%)** | |
| --- | --- | --- |
| 4 weeks (N=24) |  | 15 (62.5) |
| 16 weeks (N=69) |  | 51 (73.9) |
| 24 weeks (N=63) |  | 47 (74.6) |
| 48 weeks (N=64) |  | 46 (71.9) |
| 96 weeks (N=67) |  | 49 (73.1) |
