# Supplementary material for: Long-term effectiveness of benralizumab in severe eosinophilic asthma patients treated for 96-weeks: data from the ANANKE study
Source: Respir Res. 2023 May 20;24:135. doi: 10.1186/s12931-023-02439-w (PMC10200058; doi:10.1186/s12931-023-02439-w)
Supplement: Supplementary file 5 — Additional file 5: Table S5. Socio-demographic, clinical and laboratory characteristics, data on prior asthma medication, exacerbations, lung function, ACT score of naïve and bio-experienced patients, collected before initiating benralizumab treatment. Data were collected at the index date or during the 12 months prior to the index date and are expressed as N, mean ± SD, or median. Unless otherwise stated, data are related to N = 124 naïve patients and N = 38 bio-experienced patients. Data are expressed as N, mean ± SD, or median. Unless otherwise stated, the evaluable population included 124 naïve patients and N = 38 bio-experienced patients. [file 12931_2023_2439_MOESM5_ESM.docx]

**Supplementary table 5**

| **Characteristics at index date** | **Naïve patients (N=124)** | **Bio-experienced patients (N=38)** |
| --- | --- | --- |
| **Age (years)** | 56.6 ± 12.3 | 54.3 ± 13.7 |
| **Females** | 82 (66.1) | 17 (55.3) |
| **BMI (kg/m^2^)**  Underweight/Normal weight  Overweight  Obese  Unknown | 47 (37.9)  46 (37.1)  19 (15.3)  12 (9.7) | 11 (28.9)  16 (42.1)  7 (18.4)  4 (10.5) |
| **Age at asthma diagnosis (years) (N=123, N=38)** | 38.4 ± 16.8 | 37.8 ± 13.2 |
| **Asthma duration (years) (N=123, N=38)** | 13.5 (8.1-26.4) | 14 (8.5-20.5) |
| **SEA duration (years) (N=120, N=38)** | 1.6 (1.0-3.0) | 3.5 (1.6-6.5) |
| **Patients positive to ≥ 1 (perennial and/or seasonal) allergen** | 56 (45.2) | 21 (55.3) |
| **Comorbidities**  ≥1 current asthma-related condition  Nasal polyposis, current or past  Chronic rhinosinusitis without nasal polyposis  Gastroesophageal reflux (GERD)  Allergic conjunctivitis  Allergic rhinitis  Other (atopic dermatitis, chronic idiopatic urticary, etc.)  ≥1 current OCS-related condition  Hypertension  Osteoporosis  Diabetes mellitus type II  Cataract  Cardiovascular diseases  Other  ≥1 other ongoing comorbidities | 71 (57.3)  68 (54.8)  32 (25.8)  29 (23.4)  22 (17.7)  32 (25.8)  13 (10.5)  44 (35.5)  25 (20.2)  12 (9.7)  6 (4.8)  6 (4.8)  3 (2.4)  20 (16.1)  20 (16.1) | 20 (52.6)  18 (47.4)  11 (28.9)  10 (26.3)  4 (10.5)  6 (15.8)  2 (5.2)  20 (52.6)  13 (34.2)  5 (13.2)  3 (7.9)  4 (10.5)  4 (10.5)  11 (28.9)  7 (18.4) |
| **BEC (cells /mm^3^)**  **Total serum IgE (IU/mL) (N=69, N=22)** | 605 (440-915)  161.0 (73.3-474.0) | 550 (300-756)  307.5 (128.0-620.0) |
| **OCS users for asthma treatment**  **OCS daily (prednisone equivalent) dose (mg) (N=28, N=11)** | 30 (24.2)  8.1 (5.0-21.3) | 11 (28.9)  10 (5-25) |
| **Exacerbations during the 12 months prior to index date (N=154)**  Patients with ≥1 exacerbation of any severity (N=117, N=37)  Patients with ≥1 severe exacerbation (N=117, N=37) | 109 (93.2)  39 (33.3) | 35 (94.6)  18 (48.6) |
| **AER, any (N=117, N=37)** | 4.15 | 3.95 |
| **AER, severe (N=117, N=37)** | 0.81 | 1.51 |
| **Lung function**  Pre-BD FEV_1_ (L) (N=82, N=29)  Pre-BD FEV_1_ predicted (%) (N=83, N=30)  Pre-BD FVC (N=80, N=28)  FeNO (ppb) (N=39, N=11) | 1.8 (1.4-2.5)  72 (54-85)  2.7 (2.2-3.4)  41.0 (22.0-66.0) | 2.1 (1.4-2.4)  71 (48-84)  3.0 (2.5-3.7)  43.0 (22.0-66.0) |
| **ACT score (N=90, N=30)** | 14.0 (12.0-18.0) | 14.0 (12.0-17.0) |
