# Supplementary figures and images for: Long-term effectiveness of benralizumab in severe eosinophilic asthma patients treated for 96-weeks: data from the ANANKE study
Source: Respir Res. 2023 May 20;24:135. doi: 10.1186/s12931-023-02439-w (PMC10200058; doi:10.1186/s12931-023-02439-w)

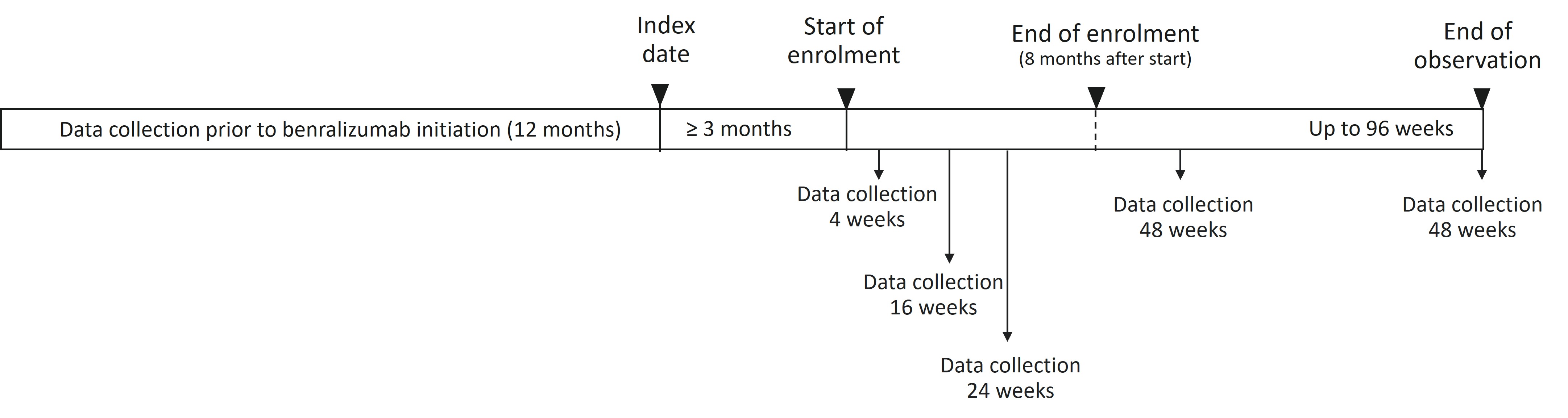

Supplement: Supplementary file 6 — Additional file 6: Figure S1. ANANKE study design. The index date represents the initiation of benralizumab treatment; enrollment of patients started at least 3 months after the index date and lasted approximately 8 months. Socio-demographic and clinical characteristics were collected at the index date and during the 12 months prior to the index date. Patients were followed up for up to 96 weeks after the index date. [file 12931_2023_2439_MOESM6_ESM.jpg]

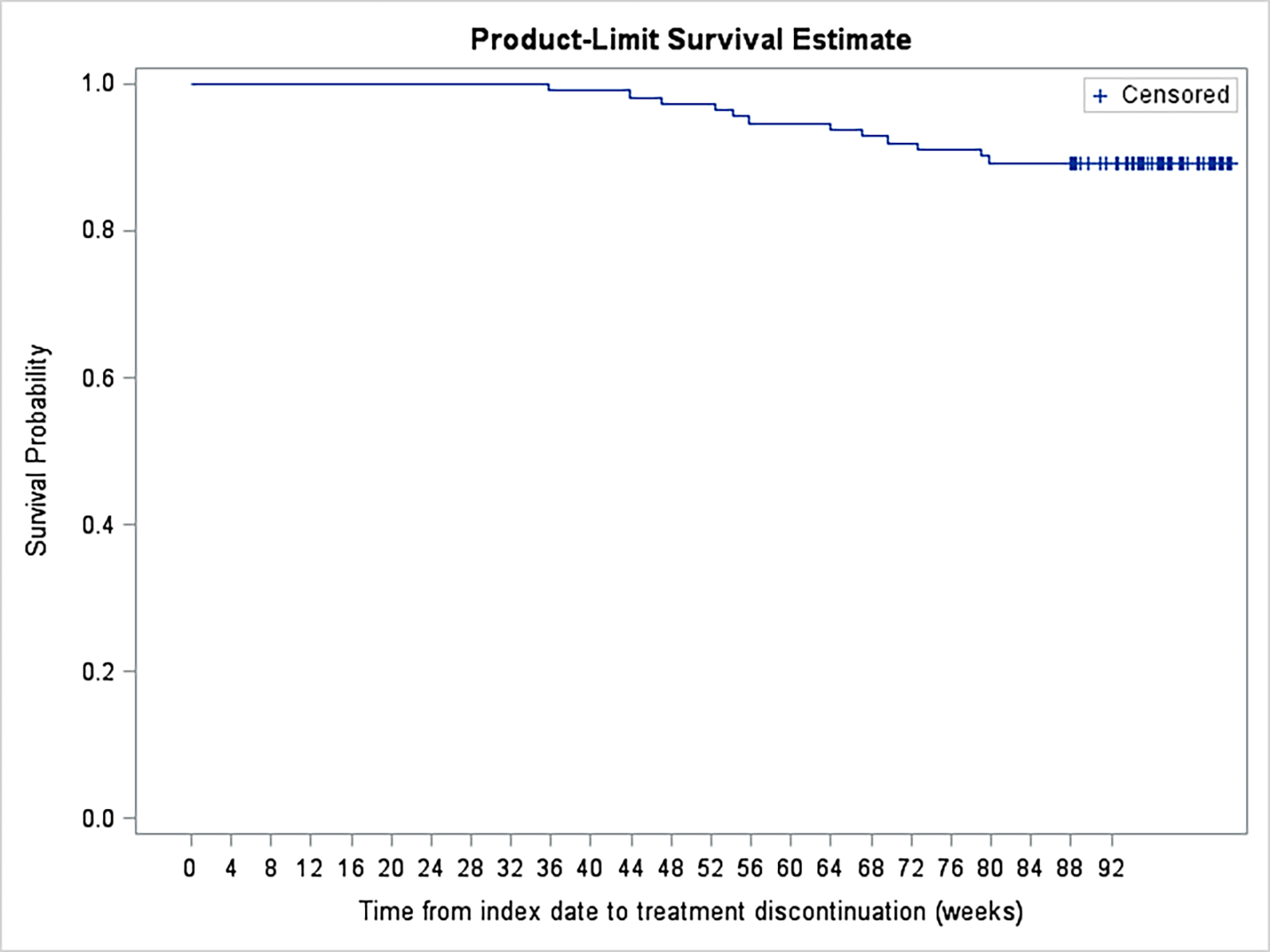

Supplement: Supplementary file 7 — Additional file 7: Figure S2. Kaplan–Meier survival analysis curve showing persistence to benralizumab treatment. For this analysis, eligible patients with consistent data were considered. Time from index date to treatment discontinuationis the time between index date and the date of benralizumab permanent discontinuation. Patients who did not interrupt benralizumab during the observation period were censored at date of enrolment visit. [file 12931_2023_2439_MOESM7_ESM.jpg]

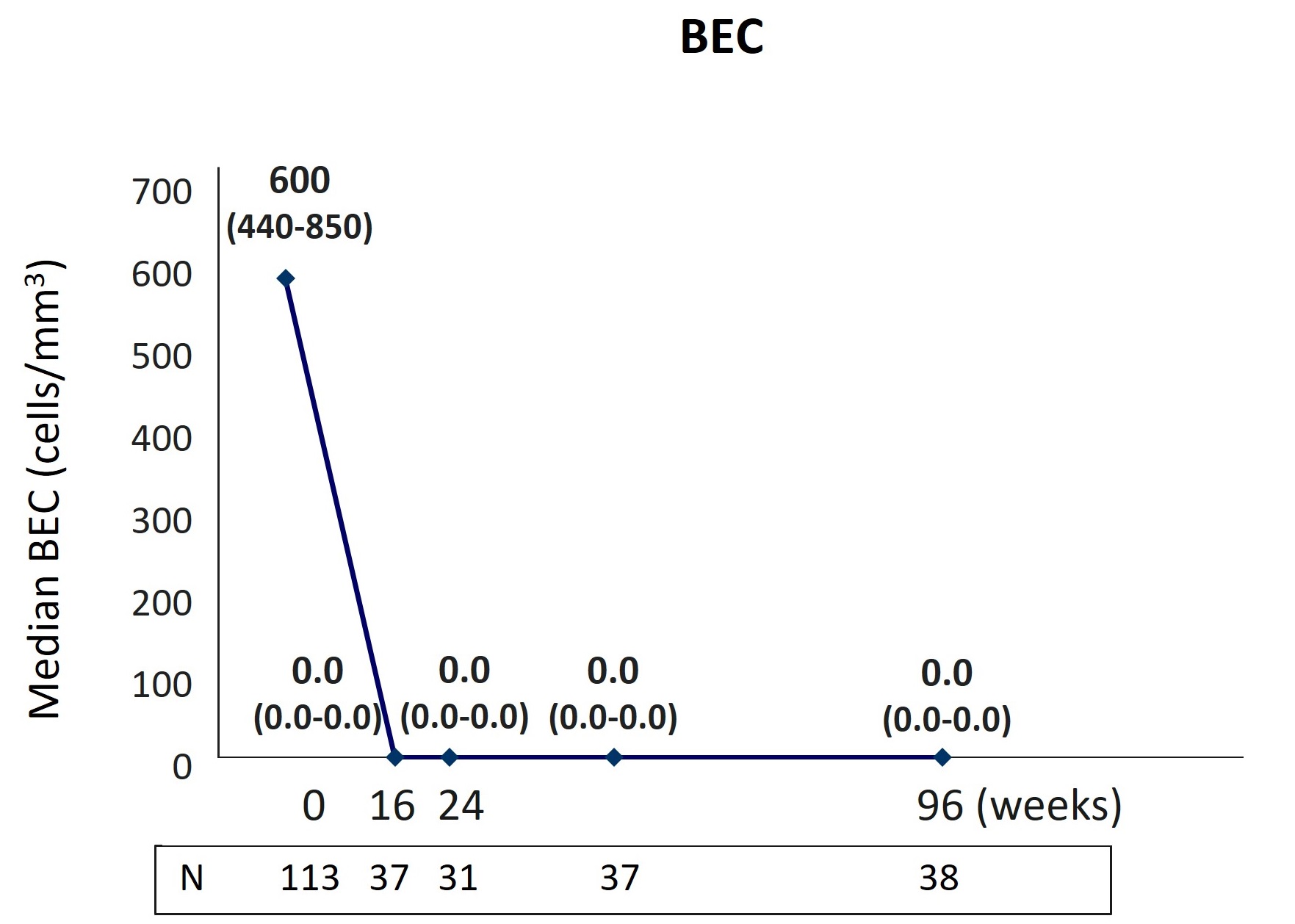

Supplement: Supplementary file 8 — Additional file 8: Figure S3. BEC depletion during benralizumab treatment. Median BECwas evaluated at index date and at 16, 24, 48 and 96 weeks. [file 12931_2023_2439_MOESM8_ESM.jpg]
